# Supplementary material for: The Construction and Exploration of a Comprehensive MicroRNA Centered Regulatory Network in Foxtail Millet (Setaria italica L.)
Source: Front Plant Sci. 2022 May 6;13:848474. doi: 10.3389/fpls.2022.848474 (PMC9121102; doi:10.3389/fpls.2022.848474)
Supplement: Supplementary file 3 [file Data_Sheet_3.zip › Sit-miR390_Seita.5G323300.1_220_TPlot.pdf]

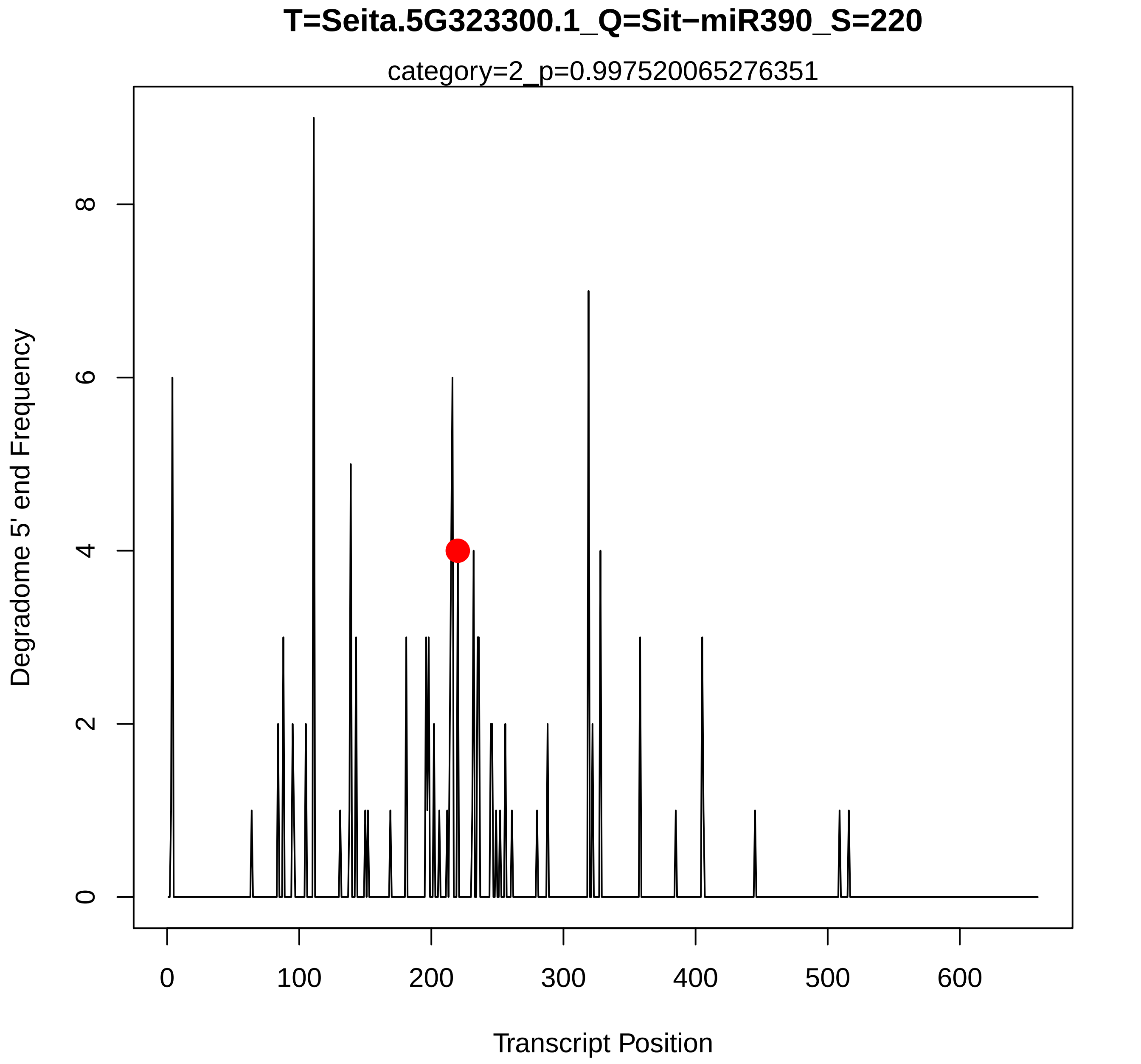
T=Seita.5G323300.1_Q=Sit−miR390_S=220
category=2_p=0.997520065276351
Degradome 5' end Frequency
0
2
4
6
8
0
100
200
300
400
500
600
Transcript Position
